# Christmas in a Yorkshire Cottage Hospital

**Published:** 1913-01-04

**Authors:** 


### CHRISTMAS IN A YORKSHIRE COTTAGE HOSPITAL.

It is Boxing Day, and everyone at the Ripon Cottage Hospital is suffering from yesterday's dissipation. I asked Augustine just now (Augustine is the cat) if he wasn't feeling chippy, and he owned up at once. If his bill of fare for the week were given, you would see how well we live in this hospital. First of all, there were the decorations. We made miles of wreathing and pricked our fingers badly, besides looking as if we had been up the chimney when we had finished. It is really surprising that holly, which appears to be red and green, and so smart and well varnished too, invariably comes off black. Then we put up our decorations, and narrowly escaped filling the beds by falling off the steps and injuring ourselves badly. Having conscientiously nailed up the holly, we saved the situation by flinging trails of ivy at the pictures and doorways, where it clung with a most graceful effect. Johnny was wishing yesterday that he lived here always, but this morning he seems rather pessimistic, and wants cold water only. I notice, too, there are other headaches in the wards.

However, we had a nice Christmas, as, I think, you only do have it in hospital. It is perhaps rather changing one form of hard work for another, but you are more than repaid by the amount of pleasure that you give to people who do not have too much in their lives. I have often heard patients say that Christmas in hospital is the happiest they have ever spent. We ask the patients' friends and our old patients to tea, and the latter say it is like coming home. After tea we had a concert, and the audience fully appreciated the efforts of the staff for their amusement. One girl prefers nurse's violin solo to anything else. "She does love the fiddle," she says. Then the doctor's comic songs bring down the house, and the choruses were enthusiastically sung. Our good vicar, too, gives another example of gratuitous work at a busy time by bringing his choir to sing carols and hymns in the evening. We feel that this touch of religious sentiment rounds off our day appropriately, as we think how differently things might have gone with some of these patients who are singing so lustily if our prayer had not been abundantly answered: "Prosper Thou the work of our hands upon us, oh prosper Thou our handiwork."

